# Activity of a Pediatric Emergency Department of a Tertiary Center in Bologna, Italy, during SARS-CoV-2 Pandemic

**DOI:** 10.3390/pediatric14030043

**Published:** 2022-08-30

**Authors:** Daniele Zama, Davide Leardini, Lorenzo Biscardi, Ilaria Corsini, Luca Pierantoni, Laura Andreozzi, Marcello Lanari

**Affiliations:** 1 Unit of Pediatrics Emergency, IRCCS Azienda Ospedaliero-Universitaria di Bologna, 40138 Bologna, Italy; 2 Department of Medical and Surgical Sciences (DIMEC), University of Bologna, 40138 Bologna, Italy; 3 Specialty School of Pediatrics, University of Bologna, 40138 Bologna, Italy

**Keywords:** SARS-CoV-2, coronavirus, pediatric emergency department, children, respiratory syncytial virus, bronchiolitis, multisystemic inflammatory syndrome

## Abstract

During the SARS-CoV-2 pandemic, the pediatric emergency department (ED) of Bologna, Emilia-Romagna, Italy faced a reorganization to better deal with the new clinical needs. We herein describe the main changes in the organization and in the attendances to our pediatric ED. From the 1 March 2020 to the 31 January 2022, 796 children positive for SARS-CoV-2 presented to our pediatric ED, but only 26 required hospitalizations, of which only 9 for COVID-19 related reasons. During this period, we also registered a temporal correlation between multisystem inflammatory syndrome in children (MIS-C) admissions and the peaks of SARS-CoV-2 infection in the Italian population. Respiratory syncytial virus (RSV) remained during last year the viral infection with the highest hospitalization rate. The analysis and description of the changes in the activity of the pediatric ED during the SARS-CoV-2 pandemic may help to better understand the routinary activity and be prepared for any possible new challenge.

## 1. Introduction

Since the beginning of the SARS-CoV-2 pandemic in March 2020, the outbreak continued to spread resulting in an unprecedented global emergency. Many countries applied preventive measures following the SARS-CoV-2 epidemiology and seasons, resulting in different ‘waves’ of COVID-19 spread related to the effect of health policy measures and the emerging of new viral variants. Since the beginning of the pandemic, Italy experienced a wide spread of the virus with more than 17 million infections, divided into four different viral outbreaks, the latest in March 2022. Regarding pediatric patients, as of 16 March 2022, in the Italian school-age population (0–19 years old), 3.072.520 positive COVID-19 cases have been registered, with 15.532 hospitalizations (0.005%), 335 admissions to the Pediatric Intensive Care Unit (PICU) and 50 deaths [1]. The Emilia-Romagna region, in the north of Italy, has been critically affected, harboring about 10% of all the Italian cases. This state of emergency and the new viral epidemiology posed a challenge for hospitals organization, especially Emergency Departments (EDs), that needed to modify their healthcare activities to ensure the correct management of infection spreading. Pediatric EDs faced a dramatic change, also considering that most of the family pediatricians could not visit patients due to the initial lack of personal protective equipment. On the other side, it has been reported a dramatic decrease in the attendance of pediatric EDs, also in Italy. The main reasons that justify this reduction were the lower prevalence of COVID-19 in children compared to the adult population, a psychological attitude toward avoiding crowded places and therefore hospitals, and a general reduction of infectious disease in the pediatric population due to the school closures and behavior changes in public health [2,3,4]. In this paper, we describe the changes in the activity during the pandemic waves in our pediatric department, the biggest tertiary pediatric center in the Emilia Romagna region in Italy.

## 2. Materials and Methods

We conducted a retrospective observational study in our pediatric ED from the 1st of March 2020 to the 31st of January 2022. Data were extracted from the electronic database of the hospital. We included all patients attending the pediatric ED for any reason, both medical and surgical. Variables extracted included the number of daily visits, the urgency codes assigned by the nurse, demographic information, the diagnosis, the positivity of the nasal swab for SARS-CoV-2 and the admission to any ward. Information regarding the national circulation of SARS-CoV-2 and cases of COVID-19 was extracted from the website of the Italian Istituto Superiore di Sanità (ISS) (https://dati-covid.italia.it; last accessed on 24 April 2022). Urgency codes in the EDs are assigned by the nurse performing the triage and are divided into high urgency codes (red codes and yellow codes) and low urgency codes (green codes and white codes). Data were represented in columns and dotted lines using Prism GraphPad 7 (San Diego, CA, USA). A comparison between the hospitalization rate in patients with bronchiolitis during the 2019–2020 and 2021–2022 seasons was performed using a Fisher exact test. The median age of the same two populations was compared with a Mann–Whitney *U* test. A comparison of the incidence of the multisystemic inflammatory syndrome (MIS-C) between the periods from November 2020 to May 2021 (period with high viral circulation) and from June 2021 to December 2021 (period with low viral circulation) was performed using the Fisher exact test. All tests were performed on SPSS version 24.0 (Armonk, NY, USA).

## 3. Results

### 3.1. Changes of the Internal Organization of the Pediatric ED

During the pandemic, the pediatric ED faced a reorganization to better deal with the infected patients and to keep track of the infections. Patients with symptoms compatible with COVID-19 (fever, respiratory symptoms, gastrointestinal symptoms, and pharyngodynia) were triaged and visited in a specific room by operators using self-protection measures. Patients without these symptoms were located in a different waiting room and had no contact with the suspected cases during the ED stay. All patients with compatible symptoms received a molecular nasal/pharyngeal swab for SARS-CoV-2 detection. All patients who required hospitalization for any reason or who had to be kept in prolonged observation received a molecular swab for SARS-CoV-2 identification. During the period from November 2020 to April 2021, all patients were also screened with rapid antigen detection kits point of care.

### 3.2. The Trend of the Attendances in ED

**Figure 1:**
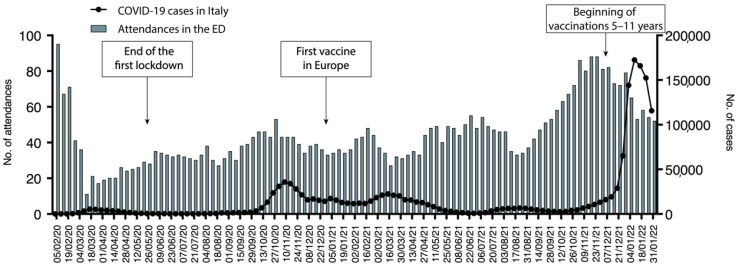
Representation of pediatric ED attendances from the beginning of the pandemic. Green columns represent the median number of daily attendances for each week. Line with dots represents the median number of COVID-19 cases for each week in Italy.

As briefly discussed, and previously described, during the SARS-CoV-2 pandemic, a dramatic decrease in the number of pediatric ED attendances was seen. The schematic representation of the trend of attendance is represented in Figure 1. We previously described during the first wave of the pandemic, in the period between the 30 January to the 31 May 2020, a 50% decrease in the admissions in our ED compared with the same period of 2019. In the period between the 10 March and the 15 April, a decrease from 2341 to 512 attendance was reported. It has been speculated that the physical distance due to the lock-down measures, better attention to hygiene, and the use of facial masks played a role in the global reduction of infectious diseases, especially in the pediatric population, and on the other hand, the fear of COVID-19 caused a decrease of non-urgent referral to pediatric ED [3].

**Figure 2:**
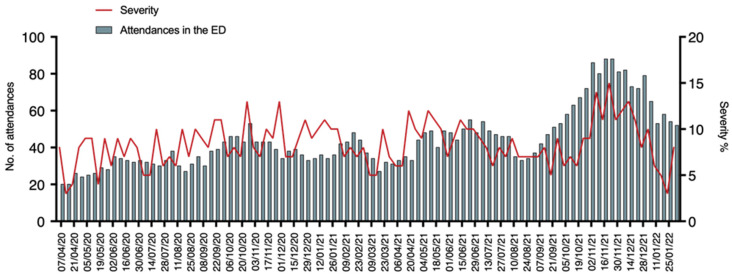
Red line represents the percentage of high urgency codes compared to the total number of accesses. Green columns represent the median number of daily attendances for each week.

During the period from April 2020 to January 2022, our pediatric ED registered a total presentation of 23,763 patients. After an initial dramatic reduction of attendances, dropping from 95 daily attendances in the first week of February 2020 to 11 during the first week of March 2020, they progressively increased. Interestingly, the lowest number of attendances were seen concurrently or short after the COVID-19 peaks. Specifically, after the peak of November 2020 pediatric ED daily attendances dropped from 43 to 34, after the peak of March 2021 from 34 to 27, after the peak of August 2021 from 46 to 35, and after the peak of January 2022 from 65 to 54. In the period from November to December 2021 attendances returned to a similar number to those before the onset of the pandemic. We then analyzed whether the pandemic changed the severity of referral to the ED. To this aim, we analyzed the number of high urgency codes (Red and Yellow Codes) compared to the total number of attendances, as represented in Figure 2.

Interestingly, the severity of the diseases referred to the ED did not show significant changes within the pandemic period with a median value of 8% of high urgency codes in the total (range 0–15%). A slight increase was seen between November and December 2021.

### 3.3. COVID-19 in Children

**Figure 3:**
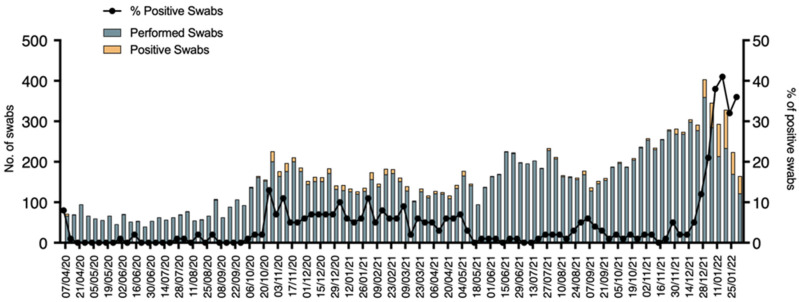
Black dots represent the percentage of positive swabs compared to the performed ones. Green columns represent the median number of daily performed swabs for each week. Yellow columns represent the median number of positive swabs for each week.

Of the 23,763 patients attending the ED from April 2020 to January 2022, 14,193 (59.7%) received a nasal/pharyngeal swab, with 796 (5.6%) resulting in positive for SARS-CoV-2 and as shown in Figure 3.

The distribution of positive clusters was seen concomitantly with national epidemical waves. The percentage of positive swabs compared to the performed one was mostly below 5% and reached higher percentages in periods with more COVID-19 spread. The percentage of positive swabs reached its peak in the period between December 2021 and January 2022 with a median of 33% positive swabs and a maximum rate of 41%. Of the 796 patients who resulted positive for COVID-19 only 26 required hospitalizations. Interestingly, only 9 patients (34%) of total admissions presented COVID-19-related symptoms, namely, 2 upper-respiratory tract infections, 1 asthma exacerbation, 2 pneumonia, 1 MIS-C, 1 difficulty in feeding, and 2 gastroenteritis. Details of the 9 patients are presented in Table 1. Other 8 (27%) patients were admitted for surgical reasons and other 8 patients (27%) for other underlying medical conditions, respectively, 1 with hemolytic anemia in G6PD-deficiency, 1 with spleen sequestration in sickle cell disease, 1 dehydration in chronic kidney disease, 1 febrile seizure, 1 with pyelonephritis, 1 morbillivirus infection, and 2 hospital admission for social needs. COVID-19 patients, who did not need hospital admission, were contacted by phone every day by our ED’s physicians until the complete resolution of the symptoms.

### 3.4. Respiratory Syncytial Virus Infection during Pandemic

**Figure 4:**
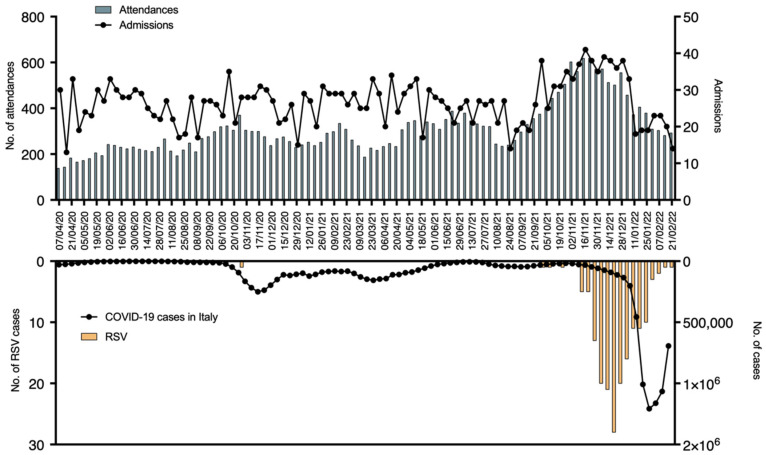
In the upper graph: line with dots represent the median number of admissions for each week and green bars the median number of attendances to the ED for each week. In the inferior graph: line with dots represents the median number of COVID-19 cases for each week in Italy and yellow bars the median number of RSV infections for each week.

As it has been already described COVID-19 pandemic also modified the epidemiology of other more common pathogens. One example is the epidemiology of Respiratory Syncytial Virus (RSV). The detailed epidemiology of RSV in our ED during the pandemic is reported In Figure 4.

Considering all the pandemic periods, the typical autumn RSV outbreak between November and March 2020 was not recorded. Only one case of RSV admission was reported in the 2020/2021 season. On the other hand, an unusual peak of RSV infections was reported in the 2021/2022 epidemic season. The peak anticipated the usual onset, being reported 13 cases between September 2021 and November 2021. Moreover, a rapid and early decrease in RSV bronchiolitis was seen, dropping from 58 cases in December 2021 to 17 in January 2022, which was concurrent with the COVID-19 spread. In fact, bronchiolitis diagnosis, as well as hospital admission, dropped as soon as COVID-19 cases increased. Interestingly, the peak of the hospitalization rate anticipated of a few weeks the peak of COVID-19 and overlapped with the RSV peak, being bronchiolitis the main cause of hospital admission in those weeks. Compared with the pre-COVID bronchiolitis season, the number of ED admissions was similar, with 229 patients in the 2019–2020 season compared with 248 in the 2021–2022 one. It is to be noted that during the last epidemic season the attendances were registered in a reduced temporal span, between September and January. During the same interval of time in 2019–2020, the bronchiolitis presentations were 40% less (151 bronchiolitis cases). RSV bronchiolitis alone accounted for up to 40% of total hospitalization causes during this period. The hospitalization rate remained similar compared to the previous epidemic season, with 45% hospitalizations in the 19–20 season and 50.4% in 2020–2021 (*p*-value 0.586). The age of patients with bronchiolitis was significantly higher in the 2020–2021 season, with a median age of 8 months (range 0–23 months), compared with the 2019–2020 one, with a median age of 6 months (range 0–13 months) (*p*-value < 0.01).

### 3.5. MIS-C Presentation during the Pandemic

**Figure 5:**
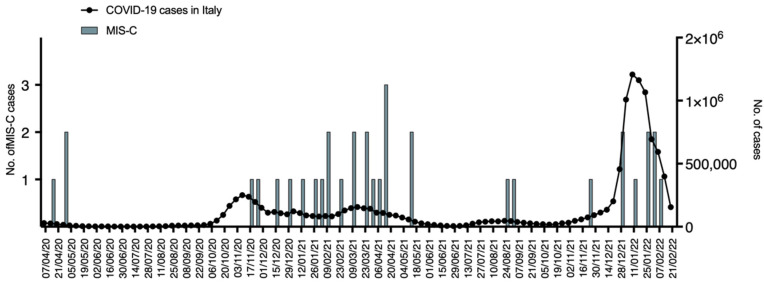
Line with dots represents the median number of COVID-19 cases for each week in Italy and green bars the median number of MIS-C infections for each week.

Other interesting data come from the new diagnosis of multisystem inflammatory syndrome in children (MIS-C), a recently described hyperinflammatory disorder associated with COVID-19. The case definition for MIS-C is an individual aged <21years old presenting with fever, laboratory evidence of inflammation, multisystem organ involvement (≥2), no alternative plausible diagnosis, and with current or recent SARS-CoV-2 infection or exposure to a suspected or confirmed COVID-19 case within the 4 weeks prior to the onset of symptoms [5,6]. In our ED during the pandemic, we diagnosed 35 cases of MIS-C. The distribution of MIS-C is presented in Figure 5.

As shown, the MIS-C cases mainly presented after the COVID-19 peaks and were less frequent in periods with a lower circulation of SARS-CoV-2. A comparison of the MIS-C incidence between periods with higher and lower prevalence of the virus revealed a statistically significant difference (Table 2).

## 4. Discussion

We herein reported the activity of a pediatric ED of a tertiary center in Italy during the SARS-CoV-2 pandemic. Analyzing the changes that occurred during a pandemic period may be a useful resource to better understand both the functioning of the ED during the non-pandemic period and the epidemiology of more common diseases. Our study presents several major results. Firstly, the SARS-CoV-2 pandemic led to an unprecedented reduction in attendances to the pediatric ED. This evidence was already reported for our hospital and for other institutions [2,3]. It can be speculated that this resulted from several conditions that changed during the whole pandemic. The preventive measures and the reduction of social contacts, especially in the first period of the pandemic, limited the circulation of both SARS-CoV-2 and other classical pathogens in the pediatric population [7]. It has been also speculated that a general fear of the health structures reduced the total number of patients attending the ED [8]. Another confirmation comes from the reductions in pediatric ED admissions we reported during and soon after COVID-19 peaks in the population (Figure 1).

Interestingly, the urgency of the conditions for referral to our ED did not differ within all the pandemic time frames. High-urgency accesses were on average 8% and low-urgency 92% of the total presentations even during the period we registered a drop in ED accesses. This may suggest that a specific social education toward appropriate use of the ED would be necessary also during pandemic periods.

Of note, the number of patients with COVID-19 that necessitated admission during the whole pandemic was extremely low suggesting that clinical manifestations were very often mild. It has to be considered that many patients referred to the pediatric ED just to assess the presence of COVID-19 even in mild symptoms presentations. It can be reasoned that a more efficient territorial health organization would have reduced the ED attendances and the correlated infectious risk in the waiting room [9]. The percentage of positive swabs was extremely variable during the whole pandemic, ranging from 0% during the low SARS-CoV-2 circulation periods to 41% during the pandemic waves (Figure 2). These data suggest the importance of testing for pathogens when more epidemiologically present in the population. It can be reasoned that specific criteria for testing for specific viruses may depend on the epidemiological periods to reduce healthcare-associated costs.

During the period between November 2020 and April 2021, Lanari et al. enrolled 1146 patients who were tested with both molecular and antigen swabs. In their study, they compared the feasibility and effectiveness of the two different methods using the data of our pediatric ED [10].

Patients who resulted positive for COVID-19 in our ED presented mostly with mild symptoms, and the vast majority (97%) did not require hospitalization. Of 26 COVID-19 positive patients who required hospitalization, only 9 presented with COVID-19 symptoms, whereas the rest were admitted for surgical or other medical reasons (Table 1). Of these 9 patients, two presented with only mild upper respiratory symptoms, and only 4 of them required a hospitalization longer than 5 days, confirming the already demonstrated data of the different clinical presentations between children and adults, with less severe presentation and lower hospitalization rate in the first population [11].

Data on RSV showed that the epidemic period 2020/2021 was totally abolished, with only one case of RSV bronchiolitis registered during the autumn season. This should be interpreted in a context of general reduction of attendances to the ED together with a change of respiratory disease epidemiology [12,13]. It is interesting to note that in the epidemic period 2021/2022, a new reappearance of RSV peak was observed, anticipating the normal mid-February peak [14]. This was also described by Camporesi et al., reporting that during the 2021–2022 bronchiolitis season in Europe and in Italy, the RSV peak was anticipated compared to usual with a shorter duration and higher case concentration. The severity however did not differ from the usual seasonal outbreak [15]. In our cohort, we confirm these observations, with several cases being reported since September 2021 and a high peak in November–December 2021, followed by a rapid decrease of RSV bronchiolitis corresponding with the fourth SARS-CoV-2 wave (Figure 4). We believe that this rapid reduction could partially be explained by the increase in hygiene standards. Moreover, hospitalization rates were similar, with no significant differences in severity from the previous outbreaks. The total number of bronchiolitis admissions was similar, but they were concentrated in a reduced period. Between September 2021 and January 2022, the number of bronchiolitis presentations almost doubled compared with the same months in 2019–2020 season. This sudden increase pushed our pediatric ED under an unexpected high-work pressure. It is interesting to note that RSV positive bronchiolitis has been the main cause of viral infection requiring hospitalization, due to respiratory failure and need for ventilation support during those weeks. The median age of children with bronchiolitis was higher during the last outbreak compared with the pre-COVID ones. The decrease in RSV circulation during the 2020–2021 season could have led to a reduced immunization in the pediatric population with consequent increase of infections also in older children after the new RSV spreading in 2021–2022 season.

Lastly, our data showed the onset of several cases of MIS-C. The symptoms that characterize this condition are systemic inflammation, fever, hypotension, and cardiac dysfunction [5]. MIS-C usually presents in geographical and temporal clusters during SARS-CoV-2 outbreaks and typically is identified after 2 to 6 weeks after the peak incidence of acute COVID-19 infection. The sample of the population attending our pediatric ED confirms this distribution with more cases being identified after SARS-CoV-2 waves compared to low viral circulation periods (Figure 5). It has been speculated that the systemic inflammation after COVID-19 can be related to the onset of this clinical presentation [16].

## 5. Conclusions

We herein reported the activity of the pediatric ED of a tertiary center in Emilia Romagna, Italy, during the pandemic. Our study presents four major insights. First, the pandemic resulted in a dramatic decrease in the attendances to the ED, while the severity of the underlying diseases did not change. Moreover, most of the COVID-19 patients showed a mild clinical course, further confirming the previous data on children and suggesting that the fear of severe diseases may have represented a cause for referral to the ED. Thirdly, RSV did not present in the epidemic period 2020–2021 and anticipated the usual onset during the following epidemic period with a higher concentration of cases, suggesting a critical role of preventive measures also on RSV spread. Lastly, MIS-C represents a novel clinical manifestation related to COVID-19 to be considered by ED physicians during COVID-19 spread periods. The main limitation of our presentation is that we reported the experience of a single center and therefore could be influenced by local variables. The analysis of clinical activities and results from the pandemic period may be useful to reorganize the usual activity of the ED and understand the epidemiology of more common and recurrent pathogens.

## Figures and Tables

**Table 1:** Clinical features of the 8 patients admitted for COVID-19 related symptoms.

	Age and Sex	Presentation Symptoms	Comorbidity/Other Medical Conditions	Days of Hospitalization	Admission Diagnosis
(1)	2 months, male	Fever, rhinorrhea	None	1 days	Upper respiratory tract infection
(2)	3 months, female	Fever	None	1 day	Upper respiratory tract infection
(3)	17 years, female	Dyspnea, fever	Asthma, grasses allergy	3 days	Asthma exacerbation
(4)	24 days, male	Fever, diarrhea	None	4 days	Gastroenteritis
(5)	1 year, male	Fever, diarrhea	None	4 days	Gastroenteritis
(6)	12 years, female	Cough, fever	21 trisomy syndrome, atrial septal defect, hypothyroidism	6 days	Pneumonia
(7)	1 month, male	Difficulty in feeding, fever	None	10 days	Difficulty in feeding
(8)	6 years, female	Fever, cough, abdominal pain	None	12 days	MIS-C
(9)	10 days, female	Apnea, difficulty in feeding	Enterobacter positive blood culture	39 days	Pneumonia

**Table 2:** MIS-C presentation during high and low COVID-19 circulation periods.

	November 2020–May 2021	June 2021–December 2021	
No. of cases of COVID-19 in Italy	3,563,570	1,761,831	
No. of attendances to our ED	8099	12,672	
No. of diagnosis of MIS-C	21	3	*p* < 0.001

## Data Availability

The datasets used and analyzed during the current study available from the corresponding author on reasonable request.
